# Health professionals’ acceptance and willingness to pay for hepatitis B virus vaccination in Gondar City Administration governmental health institutions, Northwest Ethiopia

**DOI:** 10.1186/s12913-019-4671-3

**Published:** 2019-11-05

**Authors:** Siwule Abiye, Mezgebu Yitayal, Giziew Abere, Asefa Adimasu

**Affiliations:** 1 University of Gondar Referral Hospital, College of Medicine and Health Sciences, University of Gondar, Gondar, Ethiopia; 2 Department of Health Systems and Policy, Institute of Public Health, College of Medicine and Health Sciences, University of Gondar, P. O. Box 196, Gondar, Ethiopia; 3 Department of Environmental and Occupational Health and Safety, Institute of Public Health, College of Medicine and Health Sciences, University of Gondar, Gondar, Ethiopia; 4 Department of Epidemiology and Biostatistics, Institute of Public Health, College of Medicine and Health Sciences, University of Gondar, Gondar, Ethiopia

**Keywords:** Health professionals, Willingness to pay, Hepatitis B virus, Vaccination, Gondar city administration, Ethiopia

## Abstract

**Background:**

Hepatitis B virus (HBV) infection is a global public health problem. The burden of the disease is high in low and middle income countries like Ethiopia. However, for highly vulnerable groups such as health professionals, vaccination coverage is a major issue in the developing countries where health professionals are expected to pay for vaccination. Therefore, the objective of this study was to assess health professionals’ acceptance and willingness to pay (WTP) and associated factors for vaccination against HBV.

**Methods:**

Cross-sectional study was conducted from March to April, 2017 in Gondar city administration governmental health institutions among 423 health professionals. Simple random sampling method was employed to select the study participants. Data were collected using self- administered questionnaire. Tobit model was used to analyze the determinants of WTP and the maximum amount of money the individuals might pay for HBV vaccination. *P*-value < 0.05 was considered statistically significant.

**Result:**

A total of 423 health professionals (physicians, nurses, midwives, laboratory technicians/technologists, and others) participated in the study with a response rate of 100, and 62.4% of them were willing to pay for HBV vaccination. The mean amount of money the participants might pay for HBV vaccination was 325.83 ± 283.46 ETB (US$ 14.39 ± 12.52). The study indicated that the WTP for HBV vaccination of health professionals from health centers was 179.41 ETB less compared to health professionals from hospital. The WTP for HBV vaccination of the participants who had no experience of seeing previous patients with HBV was 157.87 ETB less compared to participants who had experience of seeing previous patients with HBV. As monthly income of the study participants increased by one ETB, the WTP was increased by 0.027 ETB.

**Conclusion:**

The study revealed that the mean amount of money the participants might pay for HBV vaccination was much less than the market price for HBV vaccination. Type of workplace and experience of seeing/observing patients with HBV, and income were the predictors of WTP for HBV vaccination. Availing the vaccine with affordable cost in governmental health institutions may increase WTP of health professionals for HBV vaccination.

## Background

Hepatitis B infection is highly infectious disease caused by Hepatitis B virus (HBV) that can be acute or chronic with illness severity from asymptomatic to symptomatic degenerative disease. It is a major public health challenge in the world infecting more than 66,000 health professionals each year [1, 2]. Vaccination against Hepatitis B saves the lives of these health professionals [3]. Around 45% of the global population live in high HBV infection prevalence (> 8%) areas [1, 4]. Acute HBV has a case fatality rate of 0.5–1% [3, 5]. Worldwide, 2 billion people have evidence of past or present infection with HBV [6], and 360 million are chronic carriers of HBV surface antigen [2, 7], and more than 686,000 people die each year from its complications [8]. Overall, HBV infection reported more in lower and middle income countries [1, 2, 9] causing a significant economic burden in terms of years of life lost [2].

Health care workers (HCWs) exposed to HBV infection were reported to be about 5.9% and the risk of contracting HBV by HCWs is fourfold higher as compared to general adult population [10, 11]. A study conducted in Ras Desta and Tikur Anbessa Hospitals in Addis Ababa, Ethiopia revealed that hepatitis surface antigen was detected in 9.7% of the HCWs [12]. Studies revealed that only 20 (5.4%) respondents in a study conducted among 370 respondents in Bahir Dar city administration, North West Ethiopia [13], and 53 (12.9%) in a study conducted among 423 HCWs in Shashemene town, Ethiopia [14] took three or more doses of hepatitis B vaccine. Another study conducted on Ethiopian surgeons’ vaccination status showed that only 18.36% received the three doses of the vaccination [15].

The most effective and feasible means of prevention for HBV is vaccination and avoidance of blood and other potentially infectious fluids [1]. World Health Organization (WHO) recommends all HCWs should be vaccinated against HBV in high epidemic areas of HBV [16]. Evidences show that HBV vaccine coverage of health care workers is low [3, 5, 15, 17]; and the reasons for not being vaccinated are lack of money, lack of awareness about the availability of the vaccine, time, perception that vaccine is not important and not at risk, negligence; work load, negligence, and peer pressure [3, 10, 11, 17, 18]. Though the Ethiopian Ministry of Health and other global agencies recommend that all health professionals should be vaccinated against HBV vaccine before starting the clinical attachments during their stay in the medical school [19], a study conducted in Amhara Regional State hospitals showed that only 4% vaccine coverage and unaffordable vaccine cost was the major reason for not being vaccinated [20].

Though limited evidences are available on health professionals’ WTP for HBV vaccination, evidences are available on WTP for vaccinations against other viruses such as Dengue virus, Tick-borne Encephalitis virus, Influenza virus, and Zika virus. These evidences show that sex, age, marital status, educational status, occupation, monthly income, residence, knowledge and attitude towards the vaccine, time consumed, perceived risk, disease related knowledge, severity of the disease, peer pressure, and medical advice are factors associated with willing to pay for vaccination [21–26].

However, health workers’ acceptance and WTP for HBV vaccination and their determinants have not yet been studied. Therefore, this study aimed to assess the health workers’ acceptance and WTP for vaccination of HBV and its determinant factors.

## Methods

### Study design and setting

Institution based cross sectional study was conducted to assess the health professionals’ WTP and associated factors for vaccination against HBV. The study was conducted from April to May, 2017 in Gondar city Administration Governmental health institutions, North Gondar zone, Amhara Regional State, Ethiopia. Gondar city administration has 12 sub-city administrations with population of 300,000 and has 8 health centers (health facilities which are staffed by around 20 professionals and provide preventive, curative, inpatient and ambulatory services, treatment of common psychiatric disorders, and dental services for 15,000–25,000 people in rural areas, and around 40,000 people in urban areas) [27], and one referral hospital. There were about 1529 health professionals (medical doctors, nurses, midwives, laboratory technologists, and others) working in these health institutions.

### Study population and sampling procedures

The study population was all health professionals who were working in the hospital and health centers. All health professionals working in the hospital and health centers during the study period were included in the study whereas health professionals who were fully vaccinated for HBV, who were HBV positive, and who were critically ill were excluded from the study.

The sample size was determined using single population proportion formula by taking 50% proportion, 95% CI, 5% margin of error, and 10% non-response rate.

$$ \mathrm{N}={\left(\mathrm{Z}\upalpha /2\right)}^2\ast \mathrm{P}\ast \left(1-\mathrm{p}\right)/\mathrm{w}2=(1.96)2\ast 0.5\ast \left(1-0.5\right)/0.025=384.16 $$

The final sample size was 384.16 + 38.414 (response rate) = 422.576 ≈ 423.

The sample was allocated proportionally to the 9 health institutions. In order to select the study participants, all the 9 health facilities available in the study area were considered, the sample was allocated to each health facility proportional to the number of health professionals in each health facility, and simple random sampling technique by using lottery method was used to select the study participants from each health facility.

### Study variables

#### Response variable

The response variables in this study were acceptance and WTP for HBV vaccination. Acceptance for HBV vaccination was measured by asking the respondents: “Are you willing to pay for HBV vaccination?” The alternative options were: “Yes” or “No”. WTP was defined as the maximum amount of money that individuals were willing to pay for HBV vaccination and it was measured by the bid contingent value method. The respondents were first asked whether they would be willing to pay a specific amount (192 ETB = 7.11 USD) and then the question was repeated using a higher or lower bid value depending on the response to the first question until the response of maximum/minimum amount the health professionals willing to pay was reached.

#### Explanatory variables

The socio-demographic and economic characteristics of the study participants such as age, sex, religion, ethnicity, marital status, educational status, occupation, work place, years of experience, and monthly income were collected. The age was recorded based on the response of the respondents. The educational status was defined as the highest level of formal education completed. The occupation of study participants was classified based on the main job of the participants: medical doctor, health officer, nursing, midwifery, laboratory technology, pharmacy, anesthesia, optometry, and other (specify). The work place was categorized as hospital and health center. Monthly income of the study participants was defined as the average amount of money earned by participants each month. Other explanatory variables such as special training about HBV, medical advice about HBV, and having experience of seeing patients who have HBV were assessed using “Yes” or “No” questions. Medical advice is the provision of a formal professional opinion or guidance or recommendations from a doctor, nurse, or other healthcare professional regarding what a specific individual should or should not do to restore or preserve health.

### Data collection procedures

A pretested and standardized questionnaire was used. The tool was prepared by reviewing factors on WTP for HBV vaccination and associated factors (Additional File 1). The socio-demographic, economic, and service and knowledge related factors were addressed. The WTP tool was prepared by using the contingent valuation method (CVM). For data collection, five nurses supervised by two supervisors were assigned with the principal investigator critical follow up. The data collectors and supervisors were approached by the principal investigator and took the data collection training if they agreed to participate in the data collection process. The data collectors and supervisors were trained for 2 days on data collection procedures.

Pre-test was conducted on 42 (10% of the sample) health professionals who were advance extension students in University of Gondar who came from Gondar Zuria health centers; and necessary modification was done after pre-test. Then, in the final data collection, the study participants assisted by the data collectors filled in the questionnaire. The collected data were checked by the data collectors’ supervisors as well as the principal investigator.

### Data processing and analysis

The data were entered into Epi Info version 7 software and exported to STATA version 14 for analysis. The data was described using frequencies and means to show distribution of the WTP and associated factors. Tobit model was used to analyze the determinants of WTP and the maximum amount of money that individuals might pay for HBV vaccination. The maximum amount of money the respondents might pay is given by:

$$ \mathrm{Y}=1\ \mathrm{if}\ \mathrm{MWTP}={\upbeta}_0+{\upbeta}_{\mathrm{i}}{\mathrm{X}}_{\mathrm{i}}+\upvarepsilon >0,\mathrm{and}\ \mathrm{Y}=0\ \mathrm{if}\ \mathrm{MWTP}\le 0 $$

Where Y = WTP for HBV vaccination; MWTP = Maximum WTP; X_i_ = Explanatory variables; β_0_ = Slope; β_i_ = Coefficient; ε = error term; 1 = Success/Yes; 0 = Failure/No.

The model estimates marginal effect of an explanatory variable on the expected value of the WTP for HBV vaccination. A *p*-value = 0.05 was used to determine statistical significance.

## Results

### Socio-demographic characteristics of study participants

A total of 423 respondents participated in this study with a response rate of 100% as the study participants involved in the study were health professionals who were aware of the public health importance of HBV vaccination and supported the purpose of this study. Among the respondents, 52.2% were males, 83.9% were Orthodox Christians, and 46.8% were nurses. The mean age and monthly income of the respondents was 29.24 ± 6.09 years, and 6180.23 ± 2247.81 ETB (US$ 272.97 ± 99.28), respectively (Table 1).

**Table 1:** Socio-demographics characteristics of study participants, Gondar City Administration Governmental Health Institutions, Northwest Ethiopia, 2017 (*N* = 423)

Variable	Frequency (n)	Percentage (%)
Sex		
Male	221	52.2
Female	202	47.8
Age (year)		
21–30	329	77.8
30–40	69	16.3
> 40	25	5.9
Religion		
Orthodox	355	83.9
Muslim	52	12.3
Others	16	3.8
Work place		
Hospital	377	89.1
Health center	46	10.9
Marital status		
Single	227	53.7
Married	186	44.0
Others	10	2.3
Occupation		
Physician	91	21.5
Nurse	198	46.8
Midwife	66	15.6
Laboratory	21	5
Others	47	11.1
Educational level		
Diploma	29	6.9
First degree	327	77.3
Second degree	61	14.4
PhD and above	6	1.4
Years of Experience		
< 5 Years	257	60.8
5–10 Years	126	29.8
> 10 Years	40	9.4
Monthly income (ETB)		
< 5000.00	132	31.2
5000-10,000.00	266	62.9
> 10,000.00	25	5.9

### Health professionals’ acceptance and WTP for HBV vaccination

There were 62.4% study participants who were willing to pay for HBV vaccination. The mean amount of money the participants might pay for HBV vaccination was 325.83 ± 283.46 ETB (US$ 14.39 ± 12.52). Among 264 participants who were willing to pay for HBV vaccination, 71 (26.9%) were physicians, 122 (46.2%) nurses, 31 (11.7%) midwives, 10 (3.8%) laboratory technicians/technologists and 30 (11.4%) other health professionals. The majority (97.6%) of study participants did not take special training about HBV.

The reasons for not being willing to pay for HBV vaccination among health professionals were the perception that the HBV vaccine may not be available in the health institutions (47.8%), considering not being at risk of HBV infection (10.1%), lack of awareness about the availability of HBV vaccine (16.4%), peer pressure (8.8%), lack of time (3.8%), and the vaccination process is time consuming (9.4%) (Table 2).

**Table 2:** Acceptance and reasons for not being willing to pay for HBV vaccination, Gondar City Administration Governmental Health Institutions, Northwest Ethiopia, 2017 (N = 423)

Variable	Frequency (n)	Percentage (%)
Do you want to pay for HBV vaccination?		
Yes	264	62.4
No	159	37.6
Why do not you want to pay for HBV vaccination?		
The vaccine is not available	76	47.8
Not at risk of acquiring the virus	16	10.1
Not aware about the availability of the vaccine	26	16.4
Peer pressure	14	8.8
Lack of time	6	3.8
Vaccination process is time consuming	15	9.4
Others	6	3.8

### Factors associated with WTP for HBV vaccination

The study revealed that workplace and experience of seeing/observing previous patients with HBV and income were factors associated with WTP for HBV vaccination. The study indicated that the WTP for HBV vaccination of health professionals from health centers was 179.41 ETB less compared to health professionals from hospital. The WTP for HBV vaccination of the participants who were from health centers was 19.39% less than those respondents who were from the hospital (*P* < 0.05, average marginal effect = −.1939135). The WTP for HBV vaccination of the participants who had no experience of seeing previous patients with HBV was 157.87 ETB less compared to participants who had experience of seeing previous patients with HBV. The WTP for HBV vaccination of participants who had no experience of seeing previous patients with HBV was 17.13% less compared to participants who had experience of seeing previous patients with HBV (*P* < 0.01, average marginal effect = −.1713363). As the monthly salary of the study participants increased by one ETB, the WTP was increased by 0.027 ETB. As monthly salary of the participants increased by 1000.00 ETB, the WTP for HBV vaccination increased by 3.70% (*P* < 0.000, average marginal effect = 0.000037) (Table 3).

**Table 3:** Factors associated with WTP for HBV vaccination, Gondar City Administration Governmental Health Institutions, Northwest Ethiopia, 2017 (N = 423)

WTP	Coef.	Std. Err.	t	P > |t|	95% Conf. Interval	Marginal Error = P(dy/dx)
Age	−5.02	5.48	−0.92	0.360	−15.79, 5.75	
Work Place						
Health Center	−**179.41**	**72.14**	**−2.49**	**0.013**	**− 321.21, −37.61**	**−.1939135**
Occupation						
Nurse	− 43.69	65.15	−0.67	0.503	−171.76, 84.38	
Midwife	− 120.20	78.55	−1.53	0.127	− 274.60, 34.21	
Lab. Technicians	−35.98	106.27	−0.34	0.735	− 244.87, 172.92	
Others	42.34	78.36	0.54	0.589	− 111.70, 196.37	
Experience	−3.17	5.43	−0.58	0.560	−13.85, 7.51	
Income	**.027**	**.011**	**2.36**	**0.018**	**.005, .049**	**.000037**
Medical Advice						
No	−43.50	41.79	−1.04	0.298	− 125.65, 38.64	
Previous Illness						
No	**−157.87**	**50.59**	**−3.12**	**0.002**	**− 257.30, −58.43**	**−.1713363**
_Constant	207.82	150.64	1.38	0.168	− 88.29, 503.93	
/sigma	367.73	17.29			333.74, 401.71	

## Discussion

This study revealed that 62.4% of health professionals were willing to pay for self-paid HBV vaccination. The mean amount of money the participants were willing to pay for HBV vaccination was 325.83 ± 283.46 ETB (US$ 14.39 ± 12.52). This is much less than the market price for HBV vaccination which is around 900 ETB (US$ 33.33). Among those who were willing to pay for HBV vaccination, 8.7% participants agreed to pay below the average vaccine price which may due to unaffordable vaccine price. This could be due to the fact that health professionals are underpaid in Sub-Saharan Africa [20]. Moreover, high cost is the main obstacle for not being-vaccinated especially in low- income countries like Ethiopia where the prevalence of hepatitis B virus is high particularly among vulnerable groups (health professionals) [28]. Health institutions should avail the vaccine with affordable cost for health professionals [29].

On the other hand, in the current study, 37.6% of participants were not willing to pay for self-paid HBV vaccination. The main reasons for not willing to pay for HBV vaccination were unavailability of HBV vaccine (47.8%) from health institutions, considering not being at risk of HBV infection, lack of awareness about the availability of HBV vaccine, peer pressure, lack of time, and the vaccination process is time consuming. In this study, unavailability of the vaccine hampered acceptance for HBV vaccination in 47.8% of participants. This finding is in accordance with a study conducted in Amhara National Regional Sate hospitals where lack of access to the vaccine was the main reason for a high non-vaccination rate among health professionals [20].

The study revealed that factors such as workplace and experience of seeing/observing patients with HBV, and income were significantly associated with WTP for HBV vaccination. Health professionals who were working in the health centers had less WTP for HBV vaccination compared with those who were working in the hospital. An increased WTP for HBV vaccination was seen among who were working in the hospital could be due to an increased awareness about the risk of HBV infection, prevention methods and advantage of being vaccinated. It is very vital raising the awareness of health care workers about the importance of being vaccinated against HBV and providing adequate information about HBV vaccine is important for risky group of population [3, 30].

Similarly, a decrease in WTP for HBV vaccination was seen among study participants who had no experience of seeing/ observing patients with HBV compared with study participants who had experience of seeing/observing patients with HBV. This could be related to the perceived seriousness or severity of the disease as reported in other study on WTP for cervical cancer screening [31].

Finally, as the income of the study participants increases, the WTP for HBV vaccination also increases. This might be related to the fact that those having more income may have additional money to allocate for promotion of their health in addition to meeting their basic needs. This is in line with a study done in Pakistan on WTP for vaccination against HBV and its determinants [32] and studies conducted on WTP for different health care services in Addis Ababa [33], Malaysia [34], and Iran [35].

## Limitation of the study

The method used in this study is partial economic evaluation approach which does not show net benefit of the HBV vaccination since the study did not account for other costs and benefits of HBV vaccination. There might also be response bias in which respondents intentionally prefer lower costs of HBV vaccination to ensure that the set price is low when currently free services end. Finally, due to lack of published evidences on WTP for self-paid HBV vaccination, particularly among health professionals, comparison with previous research findings was limited. The other limitation of the study was that we could not measure the actual monthly income of the study participants which might overestimate or underestimate the WTP for HBV vaccination.

## Conclusion

The study revealed that the mean amount of money the participants were willing to pay for HBV vaccination was much less than the market price for HBV vaccination. Place of work and experience of seeing/observing patients with HBV and income were the predictors of WTP for HBV vaccination. Availing the vaccine with affordable cost in governmental health institutions such as hospitals and health centers may increase WTP of health professionals for HBV vaccination.

## Supplementary information

**Additional file 1.** Information Sheet and Consent Form, and Questionnaire on Health professionals’ willingness to pay for Hepatitis B virus vaccination in Gondar City Administration Governmental Health Institutions, Northwest Ethiopia.

## Data Availability

The datasets supporting the conclusions of this article are available upon request to the corresponding author. Due to data protection restrictions and participant confidentiality, we do not make participants data publicly available.

## Abbreviations

AOR: Adjusted Odds Ration
CVM: Contingent Valuation Method
HBV: Hepatitis B Virus
HCWs: Health Care Workers
HIV: Human Immunodeficiency Virus (HIV)
WHO: World Health Organization
WTP: Willingness to Pay
