# Supplementary material for: Health professionals’ acceptance and willingness to pay for hepatitis B virus vaccination in Gondar City Administration governmental health institutions, Northwest Ethiopia
Source: BMC Health Serv Res. 2019 Nov 5;19:796. doi: 10.1186/s12913-019-4671-3 (PMC6833239; doi:10.1186/s12913-019-4671-3)
Supplement: Supplementary file 1 — Additional file 1. Information Sheet and Consent Form, and Questionnaire on Health professionals’ willingness to pay for Hepatitis B virus vaccination in Gondar City Administration Governmental Health Institutions, Northwest Ethiopia. [file 12913_2019_4671_MOESM1_ESM.docx]

**Health Professionals’ Willingness to Pay and Associated Factors for Hepatitis B Virus Vaccination in Gondar City Administration Governmental Health Institutions, Northwest Ethiopia**

**Annex A: Information Sheet**

**Introduction:** This information sheet and consent form is prepared to explain the study you are being asked to join. Please read carefully and ask any questions about the study before you agree to join. You may ask questions at any time after joining the study. The investigator include final year health economics masters graduate student from the Institute of public health, college of medicine and health science, university of Gondar, and two advisor from the university of Gondar.

**Purpose of Research Project:** The purpose of this research is to assess the willingness to pay for hepatitis B virus vaccination and associated factors among health professionals working in Gondar city administration governmental health institutions The study will be helpful as base line information to policy makers and also will serve as a reference for subsequent studies in the country.

**Procedure:** to assess the willingness to pay for hepatitis B virus vaccination and associated factors among health professionals in Gondar city administration governmental health institutions, you are invited to participate. If you are willing to participate in this project, you need to understand and sign the agreement form. Then after, you will administer the questionnaire that the data collector gives you. You do not need to tell your name to the data collector and all your responses and the results obtained will be kept confidentially by using coding system whereby no one will have access to your response.

**Risk/ Discomfort:** By participating in this research project, you may feel that it has some discomfort especially on wasting time about 30 minutes. We hope you will participate in the study for the sake of the benefit of the research result. There is no risk in participating in this research project.

**Benefits:** If you participate in this research project, there may not be direct benefit to you but your participation is likely to help us in assessing willingness to pay for Hepatitis B virus and to identify factors influencing this willingness.

**Incentives:** You will not be provided any incentives or payment to take part in this project.

**Confidentiality:** The information collected from this research project will be kept confidential and information about you that will be collected by this study will be stored in a file, without your name, but a code number assigned to it. And it will not be revealed to anyone except the principal investigator and will be kept locked with key.

**Right to refuse or withdraw:** You have full right to refuse from participating in this research. You can choose not to respond to some or all questions if you do not want to give your response. You have also the full right to withdraw from this study at any time you wish, without losing any of your right.

**Persons to contact:** If you have any question, please contact the following persons.

1. Siwule Abiye: Phone No. 0912776296
2. Dr. Mezgebu Yitayal: Phone No. 0947057683
3. Mr. Geziew Abera: Phone No. 0918814052

**Annex B: Consent Form**

Dear participant; my name is _______________. I am working with Siwule Abiye, who is doing a research as partial fulfillment for the requirement of Master of Health Economics at University of Gondar.

The main aim of his study is to assess the willingness to pay for hepatitis B virus vaccination and to identify associated factors *.*The results of the study will be used as base line information to policy makers and planners. Your name will not be written in this form and the information you give is kept confidential. If you do not want to answer all or some of the questions, you do have the right to do so. However, your willingness to answer all of the questions would be appreciated. If you have any question, don’t hesitate to ask the data collector. However you are not expected to answer the questions if you are positive for hepatitis B test. It doesn’t take more than 30 minutes.

Would you participate in responding to the questions in this questionnaire?

Yes ________________________ No__________________________

Name and Signature of the data collector _________________________

Date of interview____________________________________________

Name and signature of the supervisor ____________________________

Date___________________________

**Thank you for your cooperation!!!**

**Annex C: English Version Questionnaire**

| Ser.  No. | Questions | Responses | Skips |
| --- | --- | --- | --- |
| 101 | How old are you? | _________________________ Years |  |
| 102 | sex | _________________________ |  |
| 103 | What is your religion? | Orthodox____________________1  Muslim______________________2  Protestant____________________3  Others (specify)_______________96 |  |
| 104 | What is your ethnicity? | Amhara______________________1  Tigrie________________________2  Oromo_______________________3  Qemant______________________4  Others (specify)_______________96 |  |
| 105 | What is your marital status? | Single_______________________1  Married______________________2  Divorced_____________________3  Widowed____________________4 |  |
| 106 | Where is your work place? | Hospital______________________1  Health Center__________________2 |  |
| 107 | What is your occupation? | Physician_____________________1  Health officer_________________2  Nurse________________________3  Midwife______________________4  Lab technician_________________5  Pharmacist____________________6  Anesthetist____________________7  Optometrist___________________8  Others (specify)_______________96 |  |
| 108 | What is your educational level? | Diploma______________________1  BSc Degree___________________2  Master’s Degree________________3  PhD and above_________________4 |  |
| 109 | Years of experience | _________________________Years |  |
| 110 | What is your monthly income | __________________________ETB |  |

**ETB = Ethiopian Birr**

**Questions on Willingness to Pay Questions and Related Factors**

**Scenario**

Hepatitis B virus is highly infectious disease and health workers are the most vulnerable groups. Despite this Hepatitis B virus is preventable and the vaccine is available. Even though Ethiopia has launched universal vaccination for children, highly vulnerable groups like health workers do not access the vaccination due to the expensiveness of the vaccine. This problem will be alleviated if both the government and the health workers share the cost of the vaccine. The questionnaire below tries to assess the willingness of the health workers to pay for the vaccination against Hepatitis B virus.

| Ser.  No. | Questions | Responses | Skips |
| --- | --- | --- | --- |
| 201 | Have you taken special training about HBV? | Yes_________________________1  No__________________________2 |  |
| 202 | Have you got any medical advice about HBV? | Yes_________________________1  No__________________________2 |  |
| 203 | Have you seen patients who have HBV? | Yes_________________________1  No__________________________2 |  |
| 204 | Do you want to pay for hepatitis B vaccination | Yes_________________________1  No__________________________2 | If “yes” skip to 206 |
| 205 | Why do not want to pay? | The vaccine is not available______1  I am not at risk of acquiring the virus _____________________________2  I am not aware that the vaccine is available______________________3  My friends are not willing to pay__4  I do not have time for vaccination__5  The vaccination process is time consumed_____________________6  The vaccine is not effective_______7  Others (specify)_______________96 |  |

| 206 | If you want will you pay 192 birr per dose | Yes_________________________1  No__________________________2 |  |
| --- | --- | --- | --- |
| 207 | What if the price is 162 birr per dose | Yes_________________________1  No__________________________2 |  |
| 208 | What if the price is 132 birr per dose | Yes_________________________1  No__________________________2 |  |
| 209 | If you really want to buy what is the final minimum amount you are willing to pay per dose vaccination | __________________________ETB | Stop the interview here |
| 210 | What if the price is 222 birr per dose | Yes_________________________1  No__________________________2 |  |
| 211 | What if the price is 252 birr per dose | Yes_________________________1  No__________________________2 |  |
| 212 | What if the price is 282 birr per dose | Yes_________________________1  No__________________________2 |  |
| 213 | If due to inflation or other uncertainties, What really is the maximum amount you are willing to pay for per dose vaccination | __________________________ETB |  |

***ETB = Ethiopian Birr***

**THANK YOU VERY MUCH!!!**
